# microRNA-1/133a and microRNA-206/133b clusters: Dysregulation and functional roles in human cancers

**DOI:** 10.18632/oncotarget.424

**Published:** 2012-02-04

**Authors:** Nijiro Nohata, Toyoyuki Hanazawa, Hideki Enokida, Naohiko Seki

**Affiliations:** ^1^ Department of Functional Genomics, Chiba University Graduate School of Medicine, Chiba, Japan; ^2^ Department of Otorhinolaryngology/Head and Neck Surgery, Chiba University Graduate School of Medicine, Chiba, Japan; ^3^ Department of Urology, Graduate School of Medical and Dental Sciences, Kagoshima University, Kagoshima, Japan

**Keywords:** cancer, miRNA, miR-1, miR-133a, miR-133b, miR-206

## Abstract

MicroRNAs (miRNAs) are endogenous short non-coding RNA molecules that regulate gene expression by repressing translation or cleaving RNA transcripts in a sequence-specific manner. A growing body of evidence suggests that miRNAs are aberrantly expressed in many human cancers and that they play significant roles in the initiation, development and metastasis of human cancers. Genome-wide miRNA expression signatures provide information on the aberrant expression of miRNAs in cancers rapidly and precisely. Recently, studies from our group and others revealed that microRNA-1 (miR-1), microRNA-133a (miR-133a), microRNA-133b (miR-133b) and microRNA-206 (miR-206) are frequently downregulated in various types of cancers. Interestingly, miR-1-1/miR-133a-2, miR-1-2/miR-133a-1, and miR-206/miR-133b form homologous clusters in three different chromosomal regions of the human genome – 20q13.33, 18q11.2 and 6p12.2, respectively. Here we review recent findings on the aberrant expression and functional significance of the miR-1/miR-133a and miR-206/miR-133b clusters in human cancers.

## INTRODUCTION

microRNAs (miRNAs) are a class of small non-coding RNA molecules consisting of 19–22 nucleotides that play important roles in a variety of biological processes, including development, differentiation, apoptosis, cell proliferation and cellular senescence [1-3].

**Figure 1:**
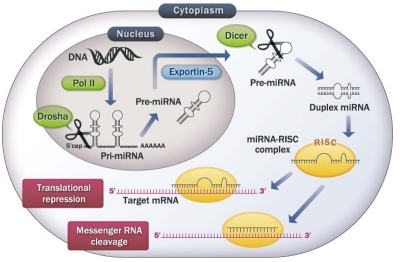
The microRNA biosynthetic pathway miRNA genes are transcribed by RNA polymerase II (Pol II). The resulting long transcript is capped with a specially-modified nucleotide at the 5' end, polyadenylated with multiple adenosines and spliced. The product is called primary miRNA (Pri-miRNA). Drosha crops Pri-miRNA into precursor-miRNA (Pre-miRNA). Pre-miRNA hairpins are exported from the nucleus to the cytoplasm by Exportin-5. In the cytoplasm, the pre-miRNA hairpin is cleaved by the RNase III enzyme Dicer. One strand is taken into the RNA-induced silencing complex (RISC), where the miRNA and its target interact. miRNAs that bind to mRNA targets with perfect matching induce mRNA cleavage, whereas translational repression is induced when matching is imperfect.

miRNAs are evolutionarily conserved and located either within the introns or exons of protein-coding genes (70%) or in intergenic regions (30%) [4]. Most intronic and exonic miRNAs are derived from their host gene, suggesting that they are transcribed concurrently with their host transcript. The others are transcribed from intergenic regions or gene deserts as separate transcriptional units [4] (Figure 1). So far, 1527 human miRNAs have been registered at miRBase in release 18.0 (http://microrna.sanger.ac.uk/). Despite the small size of these molecules through several intracellular processing [5], mature miRNAs broadly regulate gene expression through translational repression and mRNA cleavage, mainly due to the lack of a requirement for perfect sequence complementarity for target binding [6, 7] (Figure 1). Bioinformatic predictions indicate that miRNAs regulate more than 30% of protein-coding genes [8].

The importance of miRNA in cancer was first recognized when miRNA genes were found to be specifically deleted in leukemia [9]. Subsequent reports have shown that miRNAs are differentially expressed in many cancers [10]. Although the biological functions of miRNAs remain mostly unknown, many recent studies suggest that miRNAs contribute to the initiation and development of various types of cancer [11, 12]. Recent reports show that some miRNAs actually control the condition of major cancer-related signaling molecules [13], such as p53 family [13, 14], retinoblastoma (Rb) [15] and epidermal growth factor receptor (EGFR) [16]. miRNAs can be separated into two main classes: those which are oncogenic and those which are tumor suppressive. Overexpressed miRNAs can act as oncogenes by repressing tumor suppressor genes, whereas underexpressed miRNAs can function as tumor suppressors by negatively regulating oncogenes [17-19].

The miR-15a and miR-16 clusters, for example, are well known to act as tumor suppressors by targeting multiple oncogenes, including BCL2, MCL1, CCND1 and WNT3A [20], whereas the miR-17-92 cluster (also known as oncomiR-1) is recognized as oncogenic [21]. Many cluster miRNAs have been highly conserved over the course of evolution [22]. These facts indicate that miRNA clusters involve not only in normal biological process but also in development of cancers.

### Genes of the miR-1/miR-133a and miR-206/miR-133b clusters

**Figure 2:**
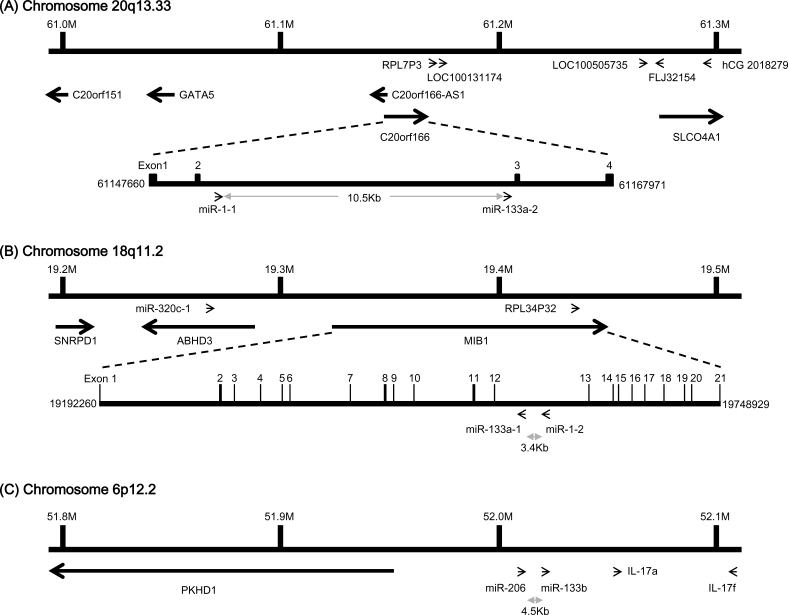
Gene structure of the human miR-1/133a and miR-206/133b clusters (A) miR-1-1 and miR-133a-2 are in an intron of the C20orf166 gene on human chromosome 20q13.33, where they are separated by 10.5kb. (B) miR-1-2 and miR-133a-1 are in a complementary strand of an intron of the MIB1 gene on human chromosome 18q11.2, where they are separated by 3.2kb. (C) miR-206 and miR-133b are clustered together on human chromosome 6p12.2, where they are separated by 4.5kb.

**Figure 3:**
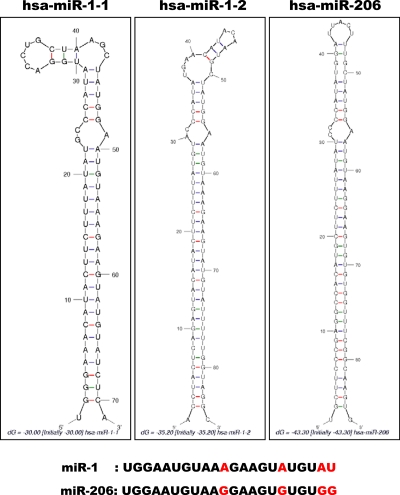
Alignment of miR-1-1, miR-1-2 and miR-206 The structures of precursor miR-1-1, miR-1-2 and miR-206 as constructed by the Mfold program [92] (http://mfold.rna.albany.edu/). The respective Pre-miRNA sequences were entered into the program. Each mature miRNA sequence is shown below with red characters indicating variant nucleotides.

**Figure 4:**
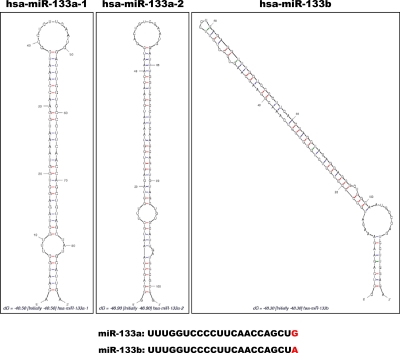
Alignment of miR-133a-1, miR-133a-2 and miR-133b The structures of precursor miR-133a-1, miR-133a-2 and miR-133b as constructed by the Mfold program [92] (http://mfold.rna.albany.edu/). The respective Pre-miRNA sequences were entered into the program. Each mature miRNA sequence is shown below with red characters indicating variant nucleotides.

Many miRNAs are expressed in a tissue-specific manner, indicating that they play important roles in many aspects of development and physiology [1, 23]. Among these are miR-1/133a and miR-206/133b, which are highly conserved in the musculatures of flies, mice and humans and are well characterized as muscle-specific miRNAs – so-called myomiRs [24, 25]. miR-1-1/miR-133a-2, miR-1-2/miR-133a-1, and miR-206/miR-133b form clusters in three different chromosomal regions in the human genome – 20q13.33, 18q11.2, and 6p12.2, respectively. miR-1-1/miR-133a-2 is in an intron of the C20orf166 gene, miR-1-2/miR-133a-1 is in an intron of the MIB1 gene, and miR-206/133b is in an intergenic region (Figure 2). miR-206 is similar to miR-1 in terms of expression and function, but its sequence differs from the miR-1 sequence by four nucleotides [26] (Figure 3). miR-133a-1 and miR-133a-2 possess identical mature sequences. miR-133b differs from miR-133a by a single nucleotide at the 3' end [26] (Figure 4).

It has been reported that the expression of miRNAs located within 50Kb of one another is highly correlated across 24 different human organs, suggesting that 50Kb might be a useful functional definition of miRNA clusters [27]. It is not rare for miRNA clusters to have paralogues or homologues in other human chromosome regions [28]. It is well known that the miR-17-92 cluster (chromosome 13q31.3) has two paralogous clusters – miR-106a-363 (chromosome Xq26.2) and miR-106b-25 (chromosome 7q22.1). miR-17-92 cluster function as oncogenes in numerous cancers [21]. On the other hand, miR-106b-25 acts as an important regulator of neural stem cell proliferation and neuronal differentiation [29]. The miR-23a~27a~24-2 cluster (chromosome 19p13.13) has one homologue – miR-23b~27b~24-1 (chromosome 9q22.32), which are related to various human health and disease status [30].

### Aberrant expression of miR-1, miR-133a, miR-133b and miR-206 in cancers

Using high-throughput technology, such as miRNA oligonucleotide arrays and quantitative RT-PCR for validation, many studies have found associations between miRNA expression levels and tumor type, grade, response to treatment and prognosis [31-36]. These studies indicate the potential for miRNAs to serve as useful markers of disease state and prognosis and predictors of drug resistance. These high-throughput analyses have found miR-1, miR-133a, miR-133b and miR-206 to be altered in various types of cancers. Except for one report about multiple myeloma [37], studies on miR-1, miR-133a, miR-133b and miR-206 have found them all to be downregulated in many types of cancer (Table 1). We and other researchers have reported that the expression levels of miR-1 and miR-133a are significantly reduced in and correlated with maxillary sinus squamous cell carcinoma (SCC), renal cell carcinoma (RCC) and rhabdomyosarcoma (RMS) [38-40].

**Table 1:** Altered expression of miR-1, miR-133a, miR-133b and miR-206 in cancers

Type of Cancer	1	133a	133b	206	Reference
**HNSCC**	**D**	**D**	**D**	**-**	[53, 74, 78, 83]
**Maxillary sinus SCC**	**D**	**D**	**-**	**-**	[38]
**Tongue SCC**	**-**	**D**	**D**	**-**	[68]
**Hypopharyngeal SCC**	**D**	**-**	**-**	**-**	[84]
**Esophageal SCC**	**D**	**D**	**D**	**-**	[69]
**Thyroid cancer**	**D**	**-**	**-**	**-**	[56, 57]
**Lung SCC**	**-**	**D**	**-**	**D**	[67]
**Lung cancer**	**D**	**-**	**D**	**D**	[43, 79]
**Gastric cancer**	**-**	**-**	**D**	**-**	[85]
**PDAC**	**-**	**D**	**-**	**-**	[86]
**HCC**	**D**	**-**	**-**	**-**	[41]
**RCC**	**D**	**D**	**-**	**-**	[39]
**Bladder cancer**	**D**	**D**	**D**	**-**	[59, 87]
**Prostate cancer**	**D**	**D**	**-**	**-**	[58, 88]
**Colorectal cancer**	**D**	**D**	**D**	**-**	[42, 70, 89-91]
**Rhabdomyosarcoma**	**D**	**D**	**-**	**D**	[40, 46, 52, 62]
**ERα positive breast cancer**	**-**	**-**	**-**	D*	[64]
**ERα positive EEC**	**-**	**-**	**-**	D*	[65]
**Multiple Myeloma**	**U**	**U**	**-**	**-**	[37]

* Compared with ERα negative cancer D: Downregulated, U: Upregulated, SCC: squamous cell carcinoma HNSCC: head and neck squamous cell carcinoma, PDAC: pancreatic ductal adenocarcinoma, HCC: hepatocellular carcinoma, RCC: Renal cell carcinoma ERα: estrogen receptor alpha, EEC: endometorial endometorioid carcinoma

It is plausible that these miRNAs are silenced by epigenetic modification. DNA methylation-mediated miR-1 silencing was suggested in hepatocellular carcinoma (HCC) after treatment with 5-aza-cytidine [41], and methylation of an miR-1-1 promoter CpG island has been found frequently in primary colorectal cancer (CRC) and colorectal adenoma [42]. In lung cancer cells, miR-1 suppression might be caused by hypoacetylation of nucleosomal histones and not DNA methylation [43].

With regard to expression levels in tumor tissues, high expression levels of miR-133b were found to be associated with poor prognosis for progression free survival in 106 patients with bladder cancer (BC) [44]. In contrast, low expression levels of miR-133b in tumor tissues were found to be associated with poor prognosis for overall survival (n=43) and positive lymph node metastasis (n=45) in CRC [45]. In RMS, low expression levels of miR-206 in tumor tissues were shown to be correlated with poor prognosis for overall survival (n=159), but no difference was found in the expression levels of miR-1 [46].

### Circulating miR-1, miR-133a, miR-133b and miR-206 as potential diagnostic markers

As noninvasive diagnostic tools, serum miRNAs have the potential to be fingerprints for diseases because human serum and plasma contain a number of stable miRNAs, and the differential expression patterns of serum miRNAs are intrinsic to a specific disease [47, 48]. miR-1 may serve as a predictor for overall survival in non-small cell lung cancer (NSCLC). Low serum expression levels of miR-1 were found to be associated with poor prognosis in NSCLC [49]. Similarly, miR-206 expression levels in serum might be used to distinguish RMS from non-RMS tumors (sensitivity of 1.0 and specificity of 0.913) [50]. In a study of gastric cancer (GC), a combination of five serum miRNAs (miR-1, miR-20a, miR-27a, miR-34 and miR-423) was a better indicator for cancer detection than conventional markers, such as CEA and CA19-9 (sensitivity of 0.8 and specificity of 0.81) [51]. Expression of these five serum miRNAs was significantly higher in GC patients. Moreover, risk score values based on the expression of the five serum miRNAs was positively correlated with clinical stage [51].

### Functional significance of miR-1, miR-133a, miR-133b and miR-206 in cancers

As miR-1, miR-133a, miR-133b and miR-206 are mostly downregulated in cancers, gain-of-function experiments are a feasible way to evaluate the functional significance of these miRNAs in various cancers.

Ectopic expression of miR-1 reportedly inhibits cell growth in HCC [41], RMS [40, 52], lung cancer [43], maxillary sinus SCC [38], head and neck squamous cell carcinoma (HNSCC) [53], laryngeal SCC [54], thyroid cancer [55-57], prostate cancer (PCa) [58], BC [59], RCC [39] and CRC [42]. miR-1 overexpression has also been reported to induce apoptosis through enhanced activation of caspases 3 and 7 and cleavage of their substrate, PARP-1, in lung cancer cells [43]. Our group also revealed that miR-1 overexpression induces apoptosis in maxillary sinus SCC [38], HNSCC [53], BC [59] and RCC cells [39] by fluorescence-activated cell sorting (FACS) analysis. FACS, TdT-mediated dUTP nick end labeling (TUNEL) and caspase assays revealed that miR-1 induces apoptosis in nasopharyngeal carcinoma cells [60]. As for cell cycle distribution, miR-1 was found to induce G0/G1 arrest in lung cancer [43], HNSCC [53], RCC [39] and RMS cells [40, 52] and G2 arrest in HCC cells [41]. Evaluations of cell migration and invasion activities have also been conducted by wound healing assay, Boyden chamber assay and invasion chamber assay. miR-1 has been found to inhibit cancer cell migration and invasion in lung cancer [43], thyroid cancer [56], HNSCC [53], laryngeal SCC [54], BC [59], RCC [39], PCa [58], RMS [52] and CRC cells [42]. In vivo, a tumor suppressive function for miR-1 was shown in lung cancer and RMS in xenotransplanted mice [43, 52].

In acute myeloid leukemia (AML) cell lines, overexpression of miR-1 promotes cell proliferation, suggesting that miR-1 might act as an oncogene in hematologic malignancy [61]. It is interesting to note that the commonalities and differences in miR-1 function depend on the type of malignant cells.

With regard to miR-206, a homologue of miR-1, the articles of its functional role are reported in RMS, breast cancer, endometrial endometorioid carcinoma (EEC) and lung cancer. Ectopic miR-206 expression inhibits cell growth in RMS [46, 52, 62], breast cancer [63, 64], EEC [65] and lung cancer cells [66]. Moreover, FACS analysis revealed that miR-206 induced apoptosis in RMS [46, 52, 62], EEC [65] and lung cancer cells [66], and induced G0/G1 arrest in RMS [46, 52, 62], breast [63] and lung cancer cells [66]. Cell migration and invasion activities are also inhibited by miR-206 in RMS [46, 52, 62], EEC [65] and lung cancer cells [66]. In RMS cells, miR-206 increased the number of myosin heavy chain (MHC)-positive cells, which means that miR-206 induced myogenic differentiation in RMS cells. Consistent with these results, miR-206 suppressed the expression of cyclin D1 and phospho-retinoblastoma protein and upregulated p21 and myogenin [62]. In vivo, a tumor suppressive function for miR-206 has been shown in RMS in xenotransplanted mice [62].

Ectopic miR-133a has been shown to inhibit cancer cell growth in lung SCC [67], maxillary sinus SCC [38], tongue SCC [68], esophageal squamous cell carcinoma (ESCC) [69], PCa [58], BC [59], RCC [39] and RMS [40], and miR-133a was found to induce apoptosis in maxillary sinus SCC [38], tongue SCC [68], BC [59], and RCC cells [39], whereas miR-133a induced G2 arrest in RCC cells [39]. Cell migration and invasion activities are also inhibited by miR-133a in ESCC [69], PCa [58], BC [59] and RCC [39]. miR-133b, a homologue of miR-133a, also inhibited tumor growth in tongue SCC [68], ESCC [69], and CRC cells [70]. Overexpression of miR-133b has been shown to induce apoptosis and G1 cell cycle arrest in CRC cells [70], whereas cell invasion activity was inhibited by miR-133b in ESCC cells [69]. In vivo, a tumor suppressive function for miR-133b was shown in CRC in xenotransplanted mice [70].

### miR-1-, miR-133a-, miR-133b- and miR-206-regulated molecular networks in cancers

Each miRNA theoretically has the potential to regulate a number of specific mRNAs, as the recognition of miRNA targets depends on the sequence complementarity of seed regions, which have lengths of about 7 nucleotides [1]. Several optimized experimental approaches can lead to identify actual miRNA target genes in a presented cell phenotype [71]. Conducting qPCR, western blotting, and reporter assays and using bioinformatic prediction programs, recent research has identified several targets of miR-1, miR-133a, miR-133b and miR-206 (Table 2). These targets potentially contribute to specific functional readouts of miR-1, miR-133a, miR-133b and miR-206. For example, the tumor suppressive function of miR-1 is partially accounted for by its repression of the oncogenic target met proto-oncogene (MET) in lung cancer [43], HCC [41], papillary thyroid cancer [57] and RMS [52]. To our knowledge, other validated oncogenic targets of miR-1 are forkhead box P1 (FOXP1) and histone deacetylase 4 (HDAC4) in lung cancer [43] and HCC [41]; LIM and SH3 protein 1 (LASP1) in BC [72]; pim-1 oncogene (PIM1) in lung cancer [43]; cyclin D2 (CCND2), chemokine (C-X-C motif) receptor 4 (CXCR4) and chemokine (C-X-C motif) ligand 12 (CXCL12) in thyroid cancer [56]; purine nucleoside phosphorylase (PNP) in maxillary sinus SCC [38] and PCa [58]; transgelin 2 (TAGLN2) in maxillary sinus SCC [38]; HNSCC [53], BC [59] RCC [39], and prothymosin alpha (PTMA) in nasopharyngeal carcinoma [60]; fibronectin 1 (FN1) in laryngeal SCC [54]; and splicing factor arginine/serine-rich 9 (SRSF9) in BC [73]. Validated targets of miR-133a are actin-related protein 2/3 complex, subunit 5 (ARPC5) in lung SCC [67]; caveolin 1 (CAV1) in HNSCC [74]; fascin homolog 1 (FSCN1) in BC [75] and ESCC [69]; glutathione S-transferase pi 1 (GSTP1) in HNSCC [76] and BC [77]; LASP1 in BC [72]; pyruvate kinase, muscle (PKM2) in tongue SCC [78]; PNP in maxillary sinus SCC [38] and PCa [58]; and TAGLN2 in maxillary sinus SCC [38] and BC [59]. miR-133b targets MET in CRC cells [70]; PKM2 in tongue SCC [68]; FSCN1 in ESCC [69] and myeloid cell leukemia sequence 1 (MCL1); and BCL2-like 2 (BCL2L2) in lung cancer [79]. MCL1 is also indirectly suppressed by miR-1 in lung cancer [43]. Target genes of miR-206 are MET in RMS [52, 62]; estrogen receptor 1 (ESR1, alias; ERα) in breast cancer [63] and EEC [65]; and notch 3 (NOTCH3) in HeLa cells [80]. As mentioned above, although the sequence of each seed region is different, some targets, such as MET, TAGLN2, PNP and LASP1, are commonly regulated by the miR-1/miR-133a and/or miR-206/miR-133b clusters. In addition, TargetScan, an miRNA target prediction program (http://www.targetscan.org/), has revealed, interestingly, that the miR-1 targets; FOXP1 and HDAC4 have putative target sites for miR-133a or miR-133b, whereas miR-133b target; BCL2L2 also has putative miR-1 or miR-206 target sites. These facts suggest that miR-1/miR-133a and miR-206/miR-133b clusters might coordinately affect downstream pathways.

**Table 2:** Validated oncogene targets of miR-1, miR-133 and miR-206 in cancers

miRNA	Symbol	Gene name	Reference
**1**	CCND2	cyclin D2	[56]
	CREB1	cAMP-responsive element binding protein 1	[55]
	CXCL12	chemokine (C-X-C motif) ligand 12	[56]
	CXCR4	chemokine (C-X-C motif) receptor 4	[56]
	FN1	fibronectin 1	[54]
	FOXP1	forkhead box P1	[41, 43]
	HDAC4	histone deacetylase 4	[41, 43]
	LASP1	LIM and SH3 protein 1	[72]
	MET	met proto-oncogene	[41, 43, 52, 57]
	PIM1	pim-1 oncogene	[43]
	PNP	purine nucleoside phosphorylase	[38, 58]
	PTMA	prothymosin alpha	[60]
	SRSF9	splicing factor arginine/serine-rich 9	[73]
	TAGLN2	transgelin 2	[38, 39, 53, 59]
**133a**	ARPC5	actin-related protein 2/3 complex, subunit 5	[67]
	CAV1	caveolin 1, caveolae protein, 22kDa	[74]
	FSCN1	fascin homolog 1, actin-bundling protein	[69, 75]
	GSTP1	glutathione S-transferase pi 1	[76, 77]
	LASP1	LIM and SH3 protein 1	[72]
	PKM2	pyruvate kinase, muscle	[68]
	PNP	purine nucleoside phosphorylase	[38, 58]
	TAGLN2	transgelin 2	[38, 39, 53, 59]
**133b**	BCL2L2	BCL2-like 2	[79]
	FSCN1	fascin homolog 1, actin-bundling protein	[69]
	MCL1	myeloid cell leukemia sequence 1 (BCL2-related)	[79]
	MET	met proto-oncogene	[70]
	PKM2	pyruvate kinase, muscle	[68]
**206**	ESR1	estrogen receptor 1	[63, 65]
	MET	met proto-oncogene	[52, 62]
	NOTCH3	notch 3	[80]

### Computational analysis of miR-1-, miR-133a-, miR-133b- and miR-206-regulated molecular networks

To reveal the biological significance of these clusters, a list of predicted targets of miR-1 or miR-206 and miR-133a or miR-133b was constructed using TargetScan (Additional Table 1). Putative miR-1 or miR-206 targets exist in 2498 genes, and putative miR-133a or miR-133b targets are found in 1756 genes. The total number of genes targeted by miR-1 or miR-206 and miR-133a or miR-133b is 3716. Common targets of miR-1 or miR-206 and miR-133a or miR-133b are 538 genes, which is 21.5% of miR-1 or miR-206 targets and 30.6% of miR-133a or miR-133b targets (Additional Table 1).

**Figure 5:**
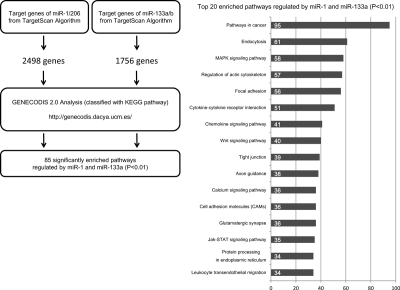
Workflow for the bioinformatic analysis of target genes of miR-1, miR-133a, miR-133b and miR-206 A total of 3716 genes were identified by the TargetScan program as predicted targets of miR-1, miR-133a, miR-133b and miR-206. The predicted target genes of miR-1 are the same as those of miR-206, and those of miR-133a are the same as those of miR-133b, due to the identical sequences of their seed regions. The genes were then analyzed and characterized in KEGG (Kyoto Encyclopedia of Genes and Genomes) pathway categories [93] by the GENECODIS program (Left). Twenty significantly enriched signaling pathways are shown in descending order of the number of genes contained in each pathway (Right).

To identify the biological processes or pathways potentially regulated by the miR-1/miR-133a and miR-206/miR-133b clusters, we performed GENECODIS analysis [81, 82] with our predicted target list. The GENECODIS analysis revealed many signaling pathways (Figure 5). Several cancers, including PCa, pancreatic cancer, lung cancer, AML, RCC, CRC, BC and thyroid cancer, are among the statistically enriched categories (Additional Table 2), and it is worth mentioning that miR-1, miR-133a, miR-133b and miR-206 are differentially expressed in these types of human malignancies. This bioinformatic analysis indicates that the miR-1/miR-133a and miR-206/miR-133b clusters might supplement each other to regulate several cancer pathways, such as cell growth, cell apoptosis, cell cycle, invasion and angiogenesis (Additional Figure 1). Thus, cooperative gene regulation by miRNAs is an interesting subject, and it may change our understanding of miRNA-mRNA interactions.

## CONCLUSION

miRNAs are important modulators of gene expression, and they contribute to a variety of biological processes, including proliferation, differentiation and apoptosis. The first evidence for their involvement in cancer was the finding in 2002 that some miRNAs are specifically deleted in leukemia. Thereafter, an enormous number of articles have been published about miRNAs and cancer. Today, nobody doubts that aberrant expression of miRNAs causes initiation, development and metastasis of human cancers.

Recently, studies from our group and others have shown that downregulation of the miR-1/miR-133a and miR-206/ miR-133b clusters are frequent events in various types of cancer. During evolution of the human genome, clustered miRNAs have developed important roles in maintaining the functions of the human body. Herein, we have reviewed the individual functions of these miRNAs and their regulated molecular targets in cooperation or independently. Elucidation of the intracellular molecular networks regulated by miRNAs is the current difficult challenge. Understanding of the molecular networks controlled by miRNA clusters may be exploited for future cancer treatment.

## Supplementary Figure and Tables






